# Supplementary material for: Parametric Amplification of Continuous-Variable Entangled State for Loss-Tolerant Quantum Distributed Sensing
Source: Sensors (Basel). 2026 Aug 31;26(17):5547. doi: 10.3390/s26175547 (PMC13568009; doi:10.3390/s26175547)
Supplement: Supplementary file 1 [file sensors-26-05547-s001.zip › sensors-4431304-supplementary.pdf]

Article

Parametric Amplification of Continuous-Variable Entangled State for Loss-Tolerant Quantum Distributed Sensing

Sijin Li * and Wei Wang *

Department of Electrical and Electronic Engineering, The Hong Kong Polytechnic University, Hong Kong 999077, China
* Correspondence: sijin.li@polyu.edu.hk (S.L.); wei-iqt.wang@polyu.edu.hk (W.W.)

1. Supplemental figures of multi-phase estimation with four-mode cluster state

Here we present various combinations of losses in four-mode cluster state and their influences on the average phase estimation sensitivities, as shown in Figs. S1-11. The results are in agreement with thoese in the main text. For example, the optimal sensitivity area is where four losses are likely symmetric in the cluster state, as can be seen, e.g. in Figs S3(a), S5(a), whether the two fixed losses are identical or not. These systematic studies of different loss configurations further validate the results concluded from the main text.

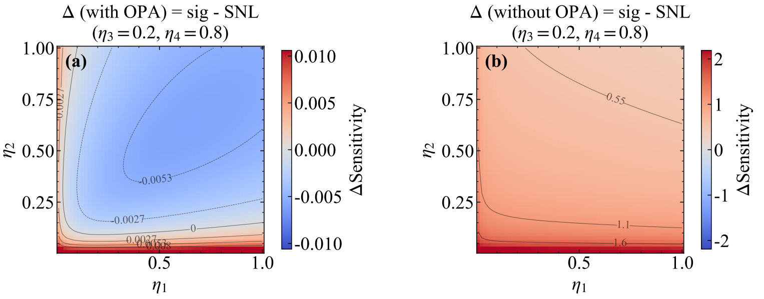

Figure S1. Average phase estimation sensitivity $\bar{\sigma}$ as a function of transmittance in modes 1 and 2, with $\eta_3 = 0.2$, $\eta_4 = 0.8$; (a) Subtraction of sensitivity with OPA and SNL; (b) Subtraction of sensitivity without OPA and SNL. Other parameters: $r_1 = 1$, corresponding to initial squeezing of about 8 dB. $r_1^{'} = 5.7$, corresponding to OPA gain of 300. $\beta_1 = \beta_2 = \beta_3 = \beta_4 = \alpha_2 = \alpha_3 = 5$, $\theta_1 = \theta_2 = \theta_3 = \theta_4 = 1.5°$.

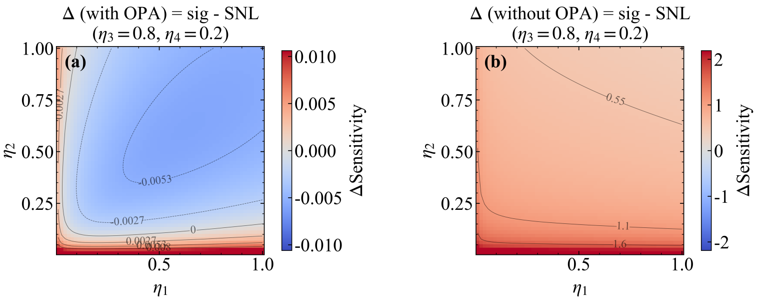

Figure S2. Average phase estimation sensitivity $\bar{\sigma}$ as a function of transmittance in modes 1 and 2, with $\eta_3 = 0.8$, $\eta_4 = 0.2$; (a) Subtraction of sensitivity with OPA and SNL; (b) Subtraction of sensitivity without OPA and SNL. Other parameters: $r_1 = 1$, corresponding to initial squeezing of about 8 dB. $r_1^{'} = 5.7$, corresponding to OPA gain of 300. $\beta_1 = \beta_2 = \beta_3 = \beta_4 = \alpha_2 = \alpha_3 = 5$, $\theta_1 = \theta_2 = \theta_3 = \theta_4 = 1.5°$.

Received: 26 June 2026
Revised: 20 August 2026
Accepted: 30 August 2026
Published: 31 August 2026

Copyright: © 2026 by the authors. Submitted to *Journal Not Specified* for possible open access publication under the terms and conditions of the Creative Commons Attribution (CC BY) license.

https://doi.org/10.3390/26175547

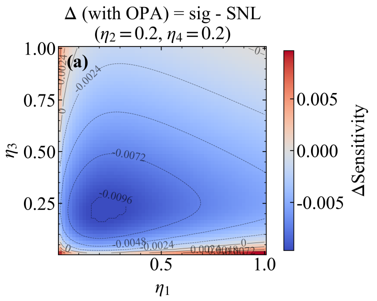

Figure S3. Average phase estimation sensitivity $\bar{\sigma}$ as a function of transmittance in modes 1 and 3, with $\eta_2 = \eta_4 = 0.2$; (a) Subtraction of sensitivity with OPA and SNL; (b) Subtraction of sensitivity without OPA and SNL. Other parameters: $r_1 = 1$, corresponding to initial squeezing of about 8 dB. $r_1^{'} = 5.7$, corresponding to OPA gain of 300. $\beta_1 = \beta_2 = \beta_3 = \beta_4 = \alpha_2 = \alpha_3 = 5$, $\theta_1 = \theta_2 = \theta_3 = \theta_4 = 1.5°$.

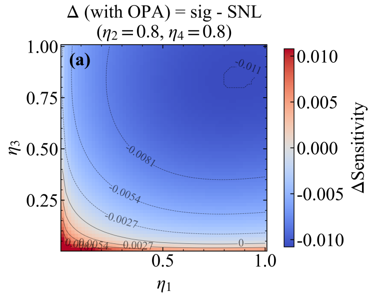

Figure S4. Average phase estimation sensitivity $\bar{\sigma}$ as a function of transmittance in modes 1 and 3, with $\eta_2 = \eta_4 = 0.8$; (a) Subtraction of sensitivity with OPA and SNL; (b) Subtraction of sensitivity without OPA and SNL. Other parameters: $r_1 = 1$, corresponding to initial squeezing of about 8 dB. $r_1^{'} = 5.7$, corresponding to OPA gain of 300. $\beta_1 = \beta_2 = \beta_3 = \beta_4 = \alpha_2 = \alpha_3 = 5$, $\theta_1 = \theta_2 = \theta_3 = \theta_4 = 1.5°$.

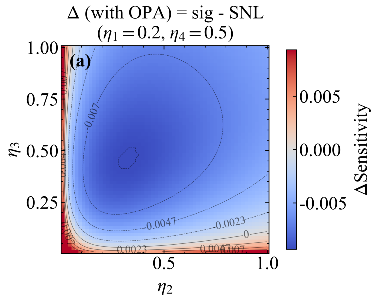

Figure S5. Average phase estimation sensitivity $\bar{\sigma}$ as a function of transmittance in modes 2 and 3, with $\eta_1 = 0.2$, $\eta_4 = 0.5$; (a) Subtraction of sensitivity with OPA and SNL; (b) Subtraction of sensitivity without OPA and SNL. Other parameters: $r_1 = 1$, corresponding to initial squeezing of about 8 dB. $r_1^{'} = 5.7$, corresponding to OPA gain of 300. $\beta_1 = \beta_2 = \beta_3 = \beta_4 = \alpha_2 = \alpha_3 = 5$, $\theta_1 = \theta_2 = \theta_3 = \theta_4 = 1.5°$.

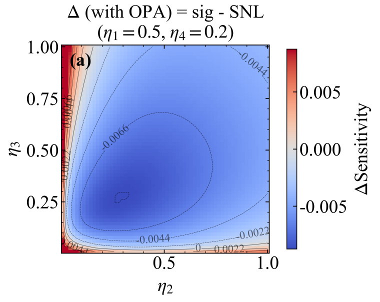

Figure S6. Average phase estimation sensitivity $\bar{\sigma}$ as a function of transmittance in modes 2 and 3, with $\eta_1 = 0.5$, $\eta_4 = 0.2$; (a) Subtraction of sensitivity with OPA and SNL; (b) Subtraction of sensitivity without OPA and SNL. Other parameters: $r_1 = 1$, corresponding to initial squeezing of about 8 dB. $r_1^{'} = 5.7$, corresponding to OPA gain of 300. $\beta_1 = \beta_2 = \beta_3 = \beta_4 = \alpha_2 = \alpha_3 = 5$, $\theta_1 = \theta_2 = \theta_3 = \theta_4 = 1.5°$.

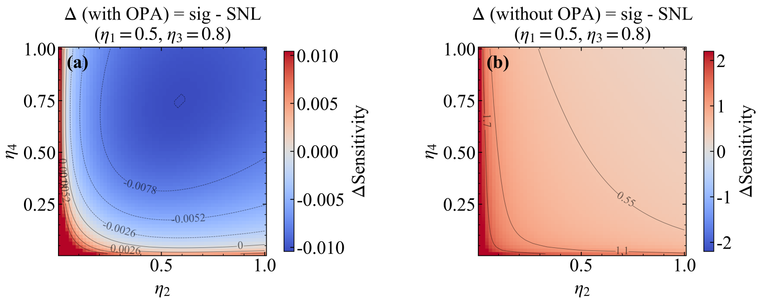

Figure S7. Average phase estimation sensitivity $\bar{\sigma}$ as a function of transmittance in modes 2 and 4, with $\eta_1 = 0.5$, $\eta_3 = 0.8$; (a) Subtraction of sensitivity with OPA and SNL; (b) Subtraction of sensitivity without OPA and SNL. Other parameters: $r_1 = 1$, corresponding to initial squeezing of about 8 dB. $r_1^{'} = 5.7$, corresponding to OPA gain of 300. $\beta_1 = \beta_2 = \beta_3 = \beta_4 = \alpha_2 = \alpha_3 = 5$, $\theta_1 = \theta_2 = \theta_3 = \theta_4 = 1.5°$.

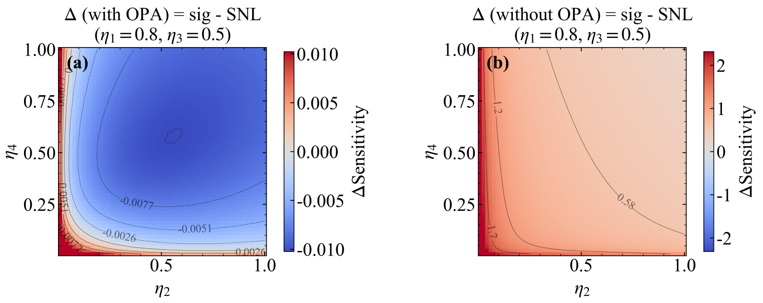

Figure S8. Average phase estimation sensitivity $\bar{\sigma}$ as a function of transmittance in modes 2 and 4, with $\eta_1 = 0.8$, $\eta_3 = 0.5$; (a) Subtraction of sensitivity with OPA and SNL; (b) Subtraction of sensitivity without OPA and SNL. Other parameters: $r_1 = 1$, corresponding to initial squeezing of about 8 dB. $r_1^{'} = 5.7$, corresponding to OPA gain of 300. $\beta_1 = \beta_2 = \beta_3 = \beta_4 = \alpha_2 = \alpha_3 = 5$, $\theta_1 = \theta_2 = \theta_3 = \theta_4 = 1.5°$.

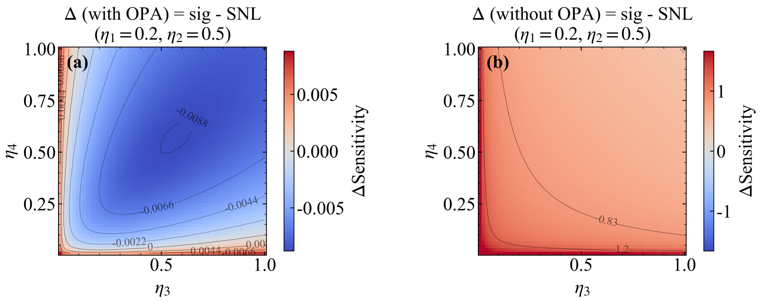

Figure S9. Average phase estimation sensitivity $\bar{\sigma}$ as a function of transmittance in modes 3 and 4, with $\eta_1 = 0.2$, $\eta_2 = 0.5$; (a) Subtraction of sensitivity with OPA and SNL; (b) Subtraction of sensitivity without OPA and SNL. Other parameters: $r_1 = 1$, corresponding to initial squeezing of about 8 dB. $r_1^{'} = 5.7$, corresponding to OPA gain of 300. $\beta_1 = \beta_2 = \beta_3 = \beta_4 = \alpha_2 = \alpha_3 = 5$, $\theta_1 = \theta_2 = \theta_3 = \theta_4 = 1.5°$.

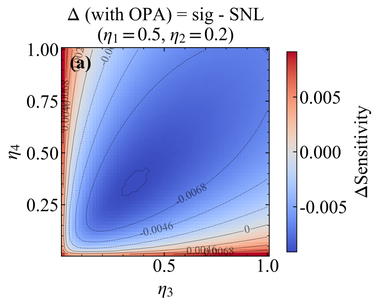

Figure S10. Average phase estimation sensitivity $\bar{\sigma}$ as a function of transmittance in modes 3 and 4, with $\eta_1 = 0.5$, $\eta_2 = 0.2$; (a) Subtraction of sensitivity with OPA and SNL; (b) Subtraction of sensitivity without OPA and SNL. Other parameters: $r_1 = 1$, corresponding to initial squeezing of about 8 dB. $r_1^{'} = 5.7$, corresponding to OPA gain of 300. $\beta_1 = \beta_2 = \beta_3 = \beta_4 = \alpha_2 = \alpha_3 = 5$, $\theta_1 = \theta_2 = \theta_3 = \theta_4 = 1.5°$.

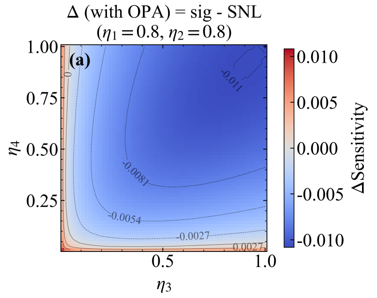

Figure S11. Average phase estimation sensitivity $\bar{\sigma}$ as function of transmittance in modes 3 and 4, with $\eta_1 = \eta_2 = 0.8$; (a) Subtraction of sensitivity with OPA and SNL; (b) Subtraction of sensitivity without OPA and SNL. Other parameters: $r_1 = 1$, corresponding to initial squeezing of about 8 dB. $r_1^{'} = 5.7$, corresponding to OPA gain of 300. $\beta_1 = \beta_2 = \beta_3 = \beta_4 = \alpha_2 = \alpha_3 = 5$, $\theta_1 = \theta_2 = \theta_3 = \theta_4 = 1.5°$.

Author Contributions: Conceptualization, S. L.; methodology, S. L.; software, S. L. and W. W.; validation, S. L. and W. W.; formal analysis, S. L.; writing—original draft preparation, S. L.; writing—review and editing, S. L. and W. W.; visualization, X.X.; supervision, S. L.; project administration, S. L.; funding acquisition, S. L. and W. W. All authors have read and agreed to the published version of the manuscript.

Funding: This research was funded by the Hong Kong Polytechnic University start-up funding, seed funding grant numbers 1-BDWT, 1-BF06, and 4-ZZYB.

Institutional Review Board Statement: Not applicable.

Informed Consent Statement: Not applicable.

Data Availability Statement: The figures and data are all numberical simulations from programs. The data and codes presented in this study are available upon request from the corresponding author.

Acknowledgments: During the preparation of this manuscript, the authors used GPT-4.1, GPT-5.1 for the purposes of editing the programming codes for simulation. The authors have reviewed and edited the output and take full responsibility for the content of this publication.

Conflicts of Interest: The authors declare no conflicts of interest.

Disclaimer/Publisher's Note: The statements, opinions and data contained in all publications are solely those of the individual author(s) and contributor(s) and not of MDPI and/or the editor(s). MDPI and/or the editor(s) disclaim responsibility for any injury to people or property resulting from any ideas, methods, instructions or products referred to in the content.
